# NDRG4 promoter hypermethylation is a mechanistic biomarker associated with metastatic progression in breast cancer patients

**DOI:** 10.1038/s41523-019-0106-x

**Published:** 2019-04-05

**Authors:** Elisa H. F. Jandrey, Ricardo P. Moura, Luciana N. S. Andrade, Camila L. Machado, Luiz Felipe Campesato, Katia Ramos M. Leite, Lilian T. Inoue, Paula F. Asprino, Ana Paula M. da Silva, Alfredo Carlos S. D. de Barros, Andre Carvalho, Vladmir C. de Lima, Dirce M. Carraro, Helena P. Brentani, Isabela W. da Cunha, Fernando A. Soares, Raphael B. Parmigiani, Roger Chammas, Anamaria A. Camargo, Érico T. Costa

**Affiliations:** 1 Centro de Oncologia Molecular, Hospital Sírio-Libanês, São Paulo, SP Brazil; 2 Ludwig Institute for Cancer Research (LICR), São Paulo, Brazil; 3 Laboratório de Oncologia Experimental, Centro de Investigação Translacional em Oncologia, Instituto do Câncer do Estado de São Paulo, São Paulo, SP Brazil; 4 Laboratório de Patologia, Hospital Sírio-Libanês, São Paulo, SP Brazil; 5 Departamento de Mastologia, Hospital Sírio-Libanês, São Paulo, Brazil; 6 Hospital do Câncer de Barretos, Barretos, SP Brazil; 7 Centro Internacional de Pesquisa, A.C. Camargo Cancer Center, Fundação Antônio Prudente, São Paulo, SP Brazil; 8 LIM23-Instituto de Psiquiatria, Faculdade de Medicina da Universidade de São Paulo (USP), São Paulo, Brazil; 9 Rede D’OR, Hospital São Luis, São Paulo, SP Brazil

## Abstract

The risk of developing metastatic disease in breast cancer patients is traditionally predictable based on the number of positive axillary lymph nodes, complemented with additional clinicopathological factors. However, since lymph node-negative patients have a 20–30% probability of developing metastatic disease, lymph node information alone is insufficient to accurately assess individual risk. Molecular approaches, such as multigene expression panels, analyze a set of cancer-related genes that more accurately predict the early risk of metastasis and the treatment response. Here, we present N-Myc downstream-regulated gene 4 (NDRG4) epigenetic silencing as a mechanistic biomarker of metastasis in ductal invasive breast tumors. While aberrant NDRG4 DNA hypermethylation is significantly associated with the development of metastatic disease, downregulation of NDRG4 transcription and protein expression is functionally associated with enhanced lymph node adhesion and cell mobility. Here, we show that epigenetic silencing of NDRG4 modulates integrin signaling by assembling β1-integrins into large punctate clusters at the leading edge of tumor cells to promote an “adhesive switch,” decreasing cell adhesion to fibronectin and increasing cell adhesion and migration towards vitronectin, an important component of human lymph nodes. Taken together, our functional and clinical observations suggest that NDRG4 is a potential mechanistic biomarker in breast cancer that is functionally associated with metastatic disease.

## Biomarker: Methylated gene promoter increases risk of metastatic disease

Epigenetic silencing of a putative tumor suppressor protein confers a metastatic advantage to breast cancer cells and could serve as a mechanistic biomarker of likely disease spread. Researchers in São Paulo, Brazil, led by Érico Costa from the Molecular Oncology Center of the Hospital Sírio-Libanês, showed that NDRG4 (short for N-Myc downstream-regulated gene 4) is actively expressed in normal breast tissue. But in breast tumors and in tumor cell lines, methyl tags cover the promoter region of the NDRG4-encoding gene, resulting in reduced protein expression, increased tumor size, elevated cell mobility and other molecular features indicative of a more aggressive disease state. The researchers experimentally silenced NDRG4 expression in two non-metastatic breast cancer cell lines and observed changes in the adhesive and migratory properties of cells that promote tumor invasiveness and spread.

## Introduction

Breast cancer patients with localized disease present a nearly 100% 5-year survival rate, but this number falls to 85% and 25% for patients with regional and distant metastasis, respectively. Indeed, distant metastases are the most life-threatening single factor in breast cancer, and the ability to predict metastatic proclivity is crucial for providing appropriate treatment and follow-up. Unfortunately, metastatic breast cancer is a terminal disease with no sustained indication of improved survival.^1,2^

As observed more than a century ago, the metastatic progression of breast cancer is not a matter of chance.^3^ Instead, specific transcriptional programs define the spreading and establishment of secondary areas of tumor growth. Recently, the identification of cancer-related genes has provided an understanding of the mechanisms that underlie malignant transformation and fostered the discovery of cancer biomarkers for early diagnosis, prognosis and disease monitoring. Moreover, newer multigene molecular panels can more accurately estimate recurrence risk and better guide improvements in adjuvant systemic therapies.^4,5^

However, despite the recent exploratory genomic studies and the discovery of novel molecular markers in breast cancer, no specific mutational drivers of metastasis have been identified. Instead, metastatic transcriptional programs have mostly emerged from epigenetic and microenvironmental optimization.^6^

Recently, a regional metastasis-specific DNA methylomes were identified for breast cancer.^7,8^ However, although a considerable number of aberrantly methylated genes have been described, the functional roles of most of these genes in malignant transformation and their potential use as cancer biomarkers have not been properly investigated. Biomarkers do not need to be directly involved in disease pathogenesis to be useful, although a biomarker is likely to be more informative if it has some mechanistic involvement. The term ‘mechanistic biomarker’ of metastasis refers to a special type of biomarker that is functionally associated with metastatic pathogenesis.

Here, we identify N-Myc downstream-regulated gene 4 (NDRG4, also known as SMAP-8 and BDM1) as a novel mechanistic biomarker of metastasis in breast tumors. NDRG4 is a 37–40 kDa intracellular protein that is predominantly expressed in the normal brain and heart.^9^ In the normal brain, NDRG4 expression protects neurons from cell death^10^ and it is dramatically decreased in the brains of Alzheimer’s disease patients in comparison to healthy brains.^9^ Molecularly, NDRG4 expressed in central nervous system (CNS) is essential for sodium channel (Na_v_) clustering at the nodes of Ranvier.^11^ In cardiac tissue, NDRG4 is critical for myocardial proliferation^12^ and the directional migration of epicardial cells on fibronectin (FN)-coated substrates.^13^ In addition, NDRG4 deregulation is an important contributor to malignant progression; however, the exact role of NDRG4 in cancer tissues remains controversial.^14–16^

In this study, we demonstrated that NDRG4 is expressed in normal breast tissue and is epigenetically silenced by DNA promoter hypermethylation in breast primary tumors and tumor cell lines. We showed that NDRG4 hypermethylation in primary breast tumors is associated with reduced NDRG4 protein expression and worse prognostic factors, such as tumor size, p53 overexpression and the presence of lymph node metastasis. Furthermore, we demonstrated that NDRG4 promoter hypermethylation is significantly associated with a lower distant metastases-free survival rate. Finally, targeting NDRG4 expression with two small-hairpin RNA (shRNA) constructs in two non-metastatic breast tumor cell lines enhanced lymph node adhesion and stimulated cell migration towards lymph node vitronectin (VN) by grouping β1-integrin receptors into large punctate clusters at the cell leading edges. Taken together, our results suggest that NDRG4 promoter hypermethylation is a promising mechanistic biomarker of metastasis in breast cancer.

## Results

### NDRG4 hypermethylation leads to gene silencing

To identify epigenetically silenced genes in an experimental breast tumor model, we hybridized RNA extracted from log-growth phase metastatic MDA-MB231 breast tumor cells treated with a methyltransferase inhibitor (5Aza-dC) with a customized cDNA microarray platform. The expression of ~1.4% of all genes analyzed (64/4608) (fold > 3) was induced by 5Aza-dC treatment (Supplementary Data 1),^17^ and 17% of the induced genes (11/64) presented a canonical CpG island located in their corresponding promoter regions (Supplementary Data 2).^17^

By interrogating this list of 11 genes using the Molecular Signatures Database (MSigDB),^18,19^ we observed significant enrichment of genes associated with integrin signaling pathways and extracellular matrix (ECM) remodeling (Supplementary Data 3).^17^ Integrins are ECM receptors that are expressed on the surface of cells that activate essential programs associated with survival, proliferation and mobility, among other processes.^20^ 5Aza-dC treatment induced the expression of integrin subunit α5 (ITGA5) and the secreted proteins plasminogen activator, urokinase (PLAU), cysteine rich angiogenic inducer 61 (CYR61) and collagen type IX α3 chain (COL9A3), which play critical roles in ECM formation and remodeling. In addition, some of the induced genes, such as CTGF^21^ and NDRG4,^13^ are known to enhance cell motility by interacting with the cytoskeleton or by modulating integrin-dependent pathways.^17^

**Fig. 1:**
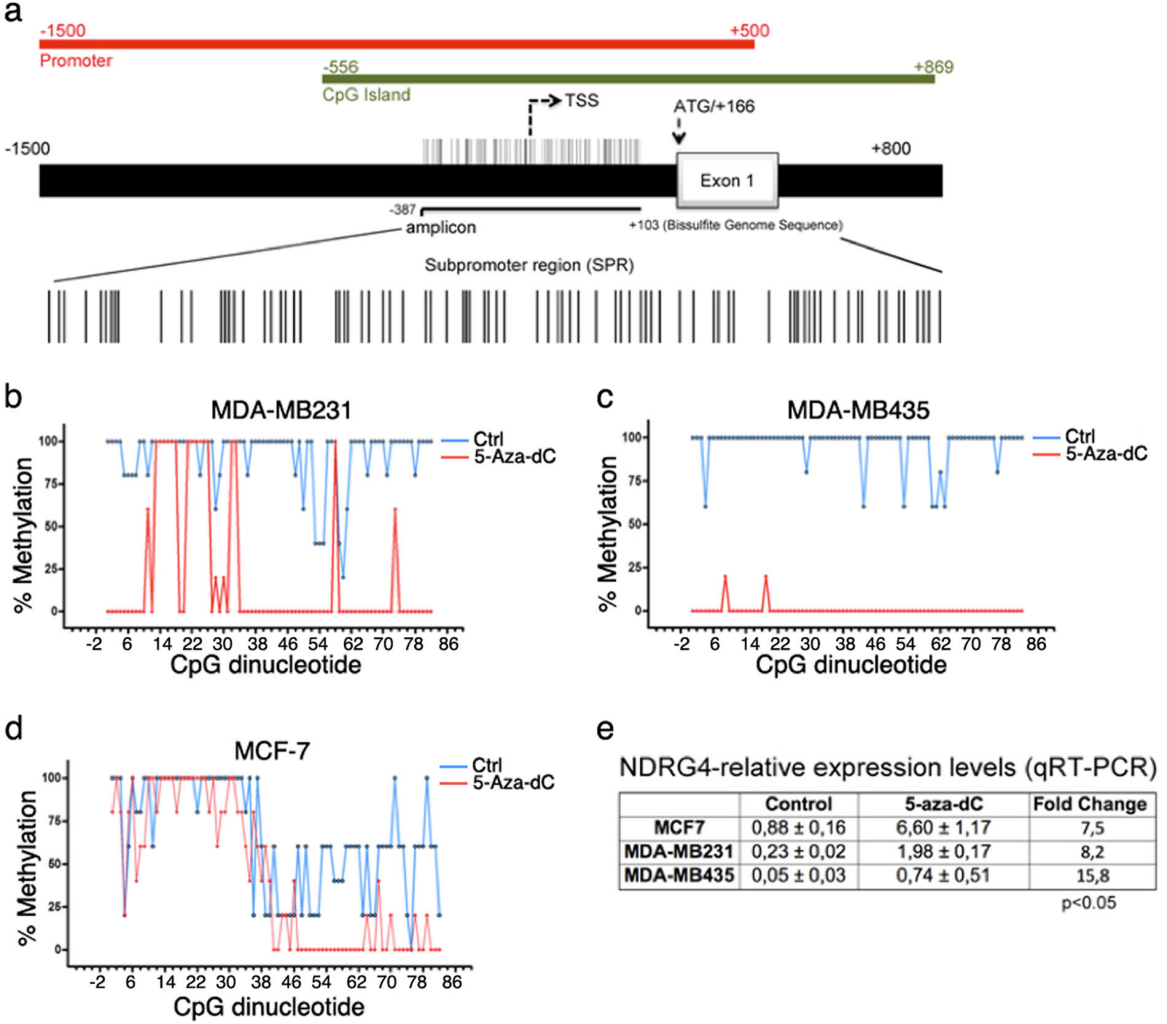
Methylation landscape and expression analysis of NDRG4 gene in breast tumor cell lines. **a** Schematic representation of NDRG4 genomic region, including promoter (positions −1500 to +500 from TSS), CpG island (−556 to +869), transcription start site (TSS) and first exon. A CpG island with 82 CGs located at positions −387 to +103 from TSS was represented in detail. **b**–**d** Percentages of methylation in these positions in two breast tumor cell lines (MCF-7 and MDA-MB231) and one melanoma cell line (MDA-MB435) before (ctrl) and 6 days after 5-aza-dC treatments (1 μM). The percentage of DNA methylation (*y*-axis) is shown as average C/T ratios. Graphs represent mean percent DNA methylation at each CpG and its position relative to TSS (*n* = 5). **e** Real-time PCR analysis of NDRG4 expression in MCF-7, MDA-MB231 and MDA-MB435 before (control) and 6 days after 5-aza-dC treatments. Expression levels relative to MCF-7 control cells and normalized to GAPDH endogenous control. Error bars represent SEM of biological replicates (*n* = 3). *p* < 0.05 by two-tailed *t* test

We decided to focus on the NDRG4 gene due to its recent validation as an epigenetic biomarker for cancer screening.^22^ In silico analysis of the NDRG4 promoter region revealed a canonical CpG island extending from nucleotide −556 to +869 relative to the NDRG4 TSS (Fig. 1a). As expected, bisulfite sequencing of a CpG-rich region located from −387 to +103 of the NDRG4 TSS confirmed a significant decrease in DNA methylation levels (from 90 to 20%) after treating the MDA-MB231 cell line with 5Aza-dC (Fig. 1b). We also observed a 15.8-fold increase in NDRG4 expression, accompanied by a 91% decrease in DNA methylation levels, in another highly metastatic cell line (MDA-MB435) after 5Aza-dC treatment. Of note, both metastatic cell lines presented lower basal NDRGA expression and higher basal methylation levels than the non-metastatic MCF-7 breast cancer cell line (Fig. 1b–e and Supplementary Fig. S1).

**Fig. 2:**
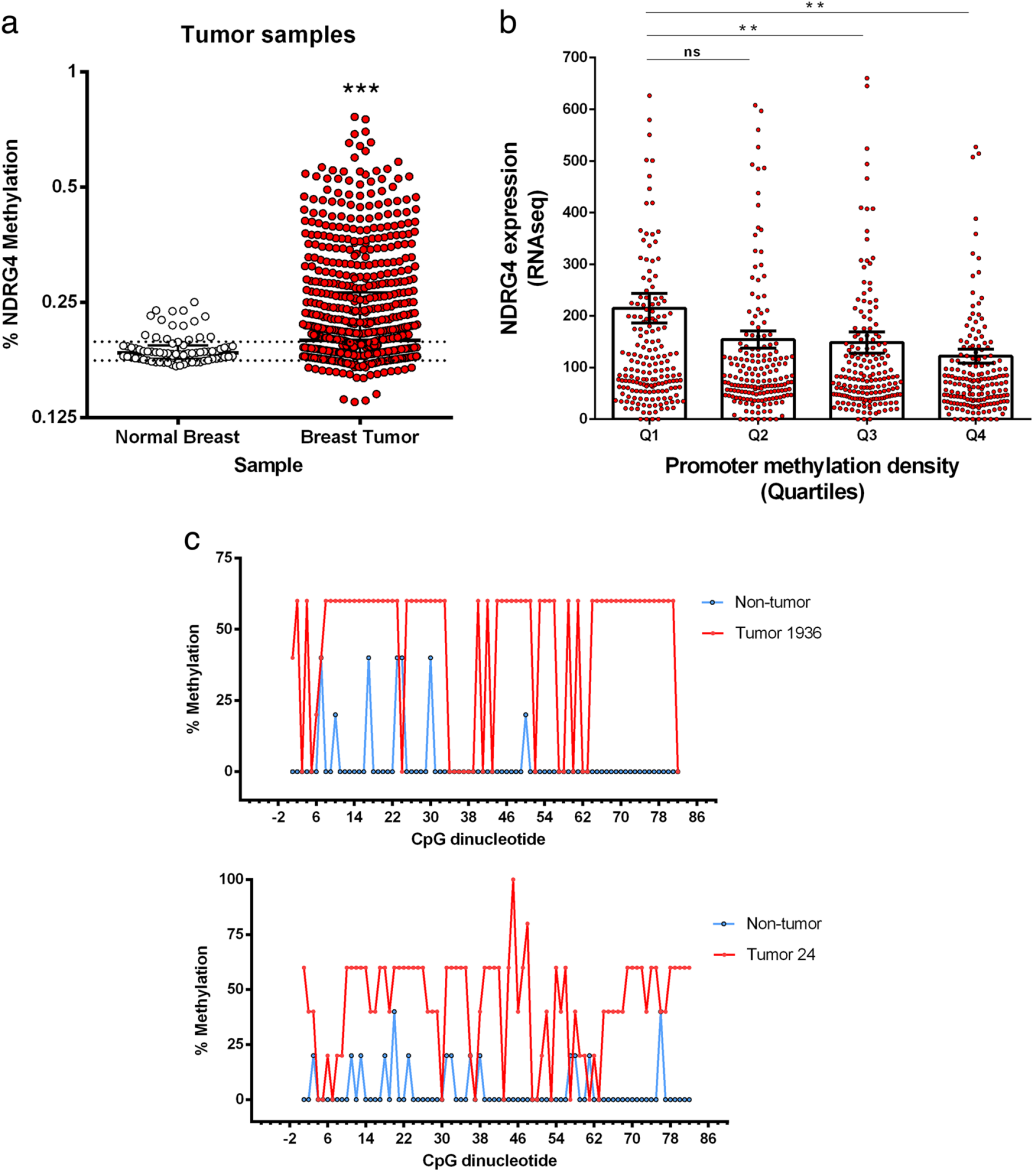
Methylation and expression analysis of NDRG4 gene in primary breast tumors (*n* = 735) and normal breast tissues (*n* = 77). **a** In silico analysis of NDRG4 promoter methylation from primary tumors and normal breast tissue from TCGA by using MethHC database. Each dot represents the percentage of NDRG4 promoter methylation. ***p < 0.001 by two-tailed *t* test. **b** MethHC database categorized the TCGA tumor samples into four classes (Q1–Q4) according to NDRG4 promoter methylation quartiles. Q1 = non-methylated. Error bars represent SEM of biological replicates. **p < 0.01, ns = not significant, by one-way ANOVA. **c** Percentages of methylation in 82 CpGs (positions −387 to +103 from TSS) in two primary breast tumors and their matched non-tumor breast tissues. Graphs represent mean percent DNA methylation at each CpG and its position relative to TSS (*n* = 5)

Next, we performed in silico expression and methylation analysis using paired normal and primary breast tumors obtained from The Cancer Genome Atlas (TCGA) through the MethHC database.^23^ On average, tumor samples exhibited higher levels of DNA methylation in the NDRG4 promoter region (−1500 to +500 relative to TSS) than their normal counterparts (Fig. 2a, *p* < 0.001).^17^ Accordingly, we categorized the TCGA tumor samples into four classes (Q1–Q4) according to NDRG4 promoter methylation quartiles. We observed a significant reduction of NDRG4 expression levels by up to 43% in the Q3 and Q4 quartiles when compared with the Q1 (non-methylated) group (p < 0.01) (Fig. 2b).^17^ In addition, breast cancer clinical stage or histological subgroups (TN, HER2+ or ER+/PR+) did not significantly differ with regard to NDRG4 promoter methylation levels (Supplementary Fig. S2). These results indicated a significant association between NDGR4 promoter methylation and reduced NDGR4 gene expression in primary breast tumors.

Consistent with these findings, bisulfite sequence analysis and nested methylation-specific PCR (Nested-MSP) showed that aberrant NDRG4 methylation is exclusively observed in primary breast tumors (Fig. 2c and Supplementary Fig. S1). These data indicate that NDRG4 promoter hypermethylation is associated with reduced NDRG4 mRNA expression in breast tumor cell lines and primary breast tumors.

### NDRG4 hypermethylation increase the risk of metastasis

**Fig. 3:**
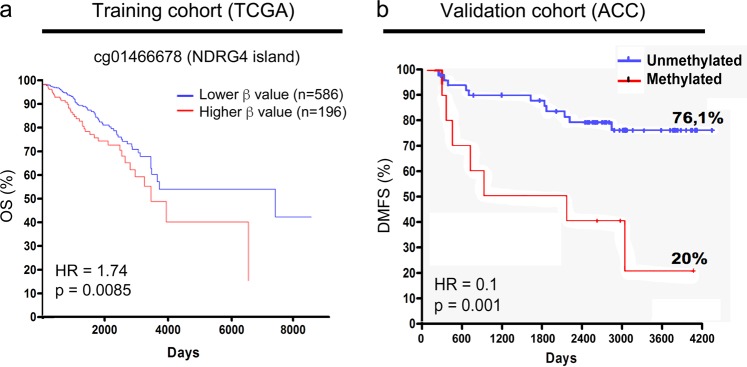
NDRG4 methylation is associated with overall survival (OS, TCGA cohort) and poor distant-metastasis free survival (DMFS, ACC cohort) in patients with breast cancer. **a** Kaplan–Meier analysis based on NDRG4 methylation levels for cg01466678 (NDRG4 promoter CpG island) by using MethSurv web tool (training cohort). *p* = 0.0085 by log-rank test. **b** Kaplan–Meier analysis based on NDRG4 DNA hypermethylation status by nested-MSP in a total of 61 ACC patient’s cohort (validation cohort) (methylated group *n* = 10, unmethylated group *n* = 51). *p* = 0.001 by log-rank test

We next explored the correlation between NDRG4 promoter methylation and expression levels and patient clinicopathological parameters and outcome. Survival analysis for the NDRG4 promoter methylation was conducted in 782 patients dichotomized in higher and lower methylation groups by MethSurv.^24^ These results significantly validate the relationship between NDRG4 promoter methylation and poor survival rate in breast cancer patients, suggesting that NDRG4 methylation may serve as a prognostic biomarker in breast cancer (cg01466678: HR = 1.7 [95%CI: 1.16–2.58], *p* = 0.0085, Fig. 3a).

To further explore previous results in an independent cohort, we examined the correlation between the presence of NDRG4 methylation and well-established prognostic factors in 61 patients with invasive breast ductal carcinoma from AC Camargo Cancer Center (ACC). The median age at the time of diagnosis was 55 years, and the median follow-up time was 120 months. Of the 61 patients analyzed, 20 (33%) patients developed distant metastases and 18 (29%) patients died during the follow-up period. Other clinicopathological data are summarized in Supplementary Data 4.^17^ NDRG4 methylation was detected in 16.4% (10/61, by nested MS-PCR) of the samples analyzed and was significantly associated with the number of positive lymph nodes (*p* = 0.025), TP53 overexpression (*p* = 0.014) and tumor size (*p* = 0.036) (Table 1). No significant association was observed between NDRG4 methylation and patient age at diagnosis, pathologic disease stage, tumor differentiation grade, expression of progesterone and estrogen receptors or lymphatic and perineural invasion (Table 1). Kaplan–Meier analysis was then performed to investigate the association between NDRG4 methylation and the development of distant metastases. Although the size of our cohort was limited to 61 patients and OS did not reach statistical significance, the 5-year distant metastasis-free survival (DMFS) rate of patients with NDRG4-methylated tumors was significantly lower than that of patients with unmethylated tumors (20% versus 76.1% respectively, HR = 0.1, *p* < 0.001, Fig. 3b and Supplementary Data 4). A multivariate analysis was then performed to determine whether NDRG4 hypermethylation was an independent factor in predicting the development of distant metastasis. All variables presenting *p* < 0.20 on the univariate analysis (Supplementary Data 4), including the use of adjuvant chemo-, radio- and hormonal therapy, were selected to build the multiple model. The number of positive lymph nodes, the use of radiotherapy, the patient age at diagnosis and NDRG4 DNA hypermethylation were shown to be independent prognostic factors for DMFS. Patients with NDRG4 DNA hypermethylation had a higher risk of developing distant metastases (HR 5.5, 95%CI 1.6–18.6, *p* = 0.006) than patients with unmethylated tumors (reference group) (Table 2). Taken together, these results suggest that NDRG4 DNA hypermethylation could be used as a complementary prognostic marker for metastatic risk in breast cancer patients.

**Table 1:** Correlation between the presence of NDRG4-SPR methylation and well-established prognostic factors in 61 patients with breast invasive ductal carcinoma

Parameters		NDRG4 promoter status		
		Meth (%)	Unmeth (%)	*p*
Age of patient (y)	<=40	8 (72.7)	3 (27.3%)	
	>40	43 (86%)	7 (14%)	0.367
Grade	1	7 (87.5%)	1 (12.5%)	
	2	31 (91.2%)	3 (8.8%)	
	3	13 (68.4%)	6 (31.6%)	0.095
SBR grade	1	13 (86.7%)	2 (13.3%)	
	2	29 (80.6%)	7 (19.4%)	
	3	8 (100%)	0	0.373
Lymph node	0	23 (88.5%)	3 (11.5%)	
involvement	1–3	18 (94.7%)	1 (5.3%)	
	>4	10 (62.5%)	6 (37.5%)	0.025
ER	Neg	18 (81.8%)	4 (18.2%)	
	Pos	33 (84.6%)	6 (15.4%)	1
PR	Neg	35 (85.4%)	6 (14.6%)	
	Pos	16 (80%)	4 (20%)	0.716
p53	Neg	38 (92.7%)	3 (7.3%)	
	Pos	11 (64.7%)	6 (35.3%)	0.014
Initial tumor size	<2	18 (94.7%)	1 (5.3%)	
(cm)	2–5	30 (83.3%)	6 (16.7%)	
	>5	3 (50%)	3 (50%)	0.036
Lymphatic	Neg	15 (93.8%)	1 (6.3%)	
Invasion	Pos	24 (72.7%)	9 (27.3%)	0.199
Perineural	Neg	30 (85.7%)	5 (14.3%)	
Invasion	Pos	17 (77.3%)	5 (22.7%)

**Table 2:** Multivariate analysis for distant metastasis-free survival

Parameters		HR	95% CI	*p*
Age of patient (y)	≤40	1.0	Ref.	–
	>40	0.1	0.04–0.4	0.001
Lymph node	0	1.0	Ref.	–
involvement	1–3	3.4	0.7–15.3	0.112
	>4	8.0	2.3–28.4	0.001
Radiotherapy adjuvant (RT)	No	1.0	Ref.	–
	Yes	0.1	0.03–0.4	0.001
NDRG4 methylation status	Neg	1.0	Ref.	–
	Pos	5.5	1.6–18.6	0.006

### NDRG4 alters the adhesive and migratory properties of cells

**Fig. 4:**
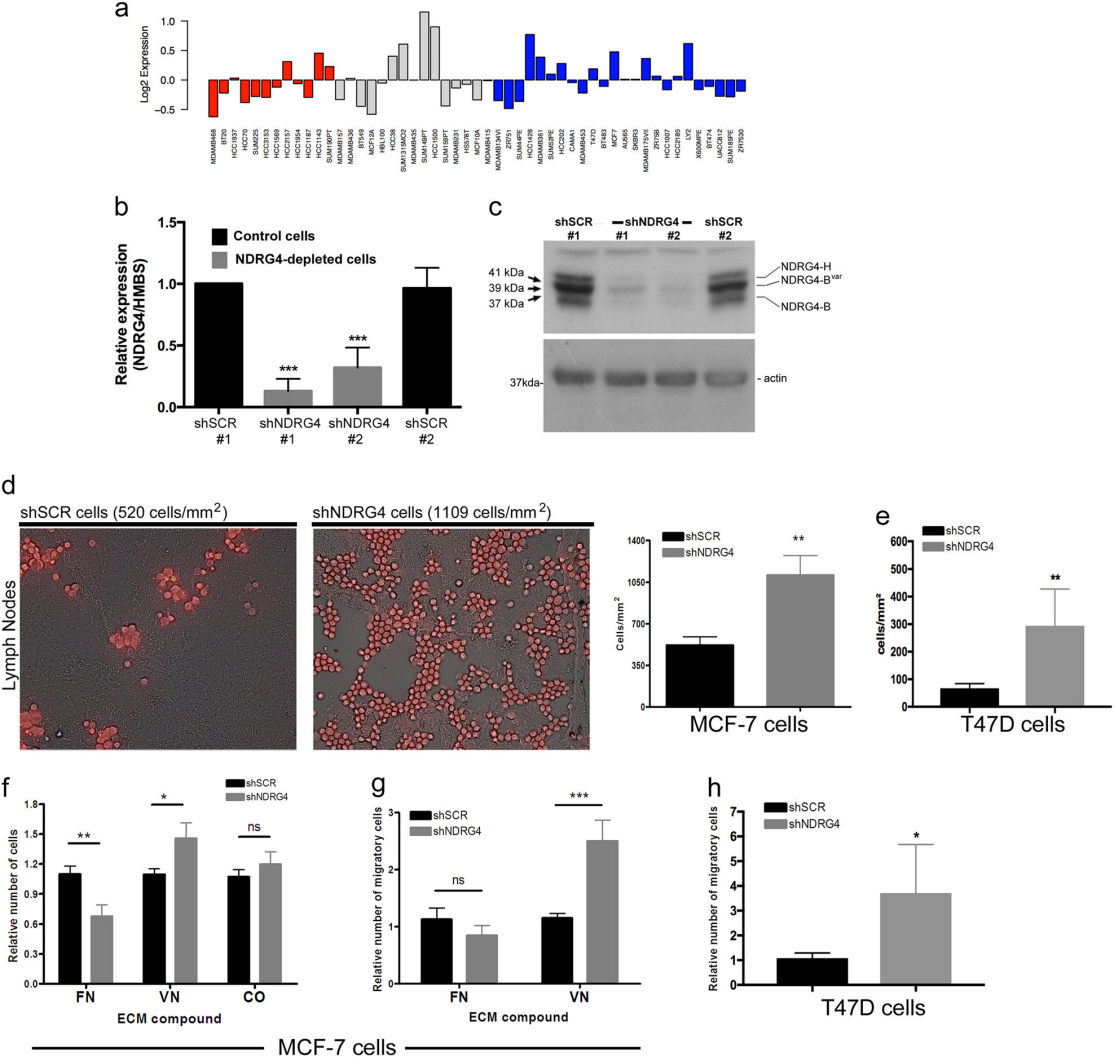
NDRG4 depletion in breast tumor cells increases lymph node adhesion and cell migration toward VN. **a** NDRG4 expression meta-analysis in human breast cancer cell lines using GOBO application^26^. Breast cancer subtypes can be identified by different colors: red = Basal A, gray = Basal B and blue = Luminal cells. **b** Analysis of NDRG4 mRNA expression levels in MCF-7 cells transfected with NDRG4-shRNAs or scramble control (shSCR). Expression levels are relative to wild type cells and normalized to hydroxymethylbilane synthase (HMBS) gene. Error bars represent SEM of biological replicates (*n* = 5). ***p < 0.001, ns = not significant, by one-way ANOVA. **c** Western blot analysis of NDRG4 expression in cytoplasmic extracts of MCF-7 shNDRG4s or shSCRs cells, using antibodies against NDRG4 and Actin. MCF-7 cells express all the three isoforms of NDRG4: 37 kDa (NDRG4-B), 39 kDa (NDRG4-B^var^) and 41 kDa isoform (NDRG4-H). For simplicity, results obtained for two independent shNDRG4 clones were grouped and presented as the shNDRG4 group. (**d**, left) Representative images of adherent red fluorescent MCF-7 cells on frozen rat lymph node sections. **d**, **e** Quantification of adherent MCF7 (**d**) and T47D (**e**) breast tumor cells in lymph nodes sections (right, *n* = 4 independent experiments for MCF-7 and 3 for T47D, **p < 0.01 by two-tailed *t* test). **f** Stationary adhesion assays showed that NDRG4 knockdown promotes ‘adhesive switch’ between FN and VN. Error bars represent SEM of biological replicates (*n* = 4). Cells do not adhere to albumin control (BSA, data not shown). *p < 0.01, **p < 0.01, ns = not significant by two-tailed *t* tests. **g**, **h** Haptotactic cell migration toward VN was analyzed by transwell migration assay in MCF-7 (**g**) and T47D (**h**) cell lines. Error bars represent SEM of biological replicates (*n* = 4). ***p < 0.001 by two-tailed *t* tests

We next decided to evaluate whether NDRG4 gene silencing has mechanistic relevance during breast cancer progression. To select a breast cancer cell line to study the role of NDRG4 silencing, we performed an expression meta-analysis in 51 human breast cancer cell lines^25^ using the GOBO application.^26^ Approximately one-quarter of the cell lines expressed a high level of NDRG4; we did not observe significant associations between NDRG4 expression levels and clinical (data not shown) or histological subgroups (represented by different colors in Fig. 4a).

In normal tissues, NDGR4 have three major variants—two are preferentially expressed in brain (B and B^var^) and one in heart (H).^9^ We chose the MCF-7 breast tumor cell line since it expresses high levels of all three NDRG4 protein isoforms (Fig. 4c) and retains several characteristics associated with a poorly aggressive disease.^27^

After knocking down NDRG4 in MCF-7 cells using two distinct shRNA sequences (named shNDRG4 cells, Fig. 4b, c), we did not perceive significant differences in growth curves in vitro or in the incidence of tumors or tumor growth in orthotopic mouse models (Supplementary Fig. S3), indicating that NDRG4 silencing has no significant effect on tumorigenesis or cell proliferation in vitro and in vivo. Similarly, the poor invasive behavior of the MCF-7 cell line cultured as 3D spheroids was not affected by NDRG4 knockdown (data not shown). However, adhesion assays using frozen lymph node sections suggest that shNDRG4 cells can attach more efficiently to lymph node matrix than control cells (1109 cells/mm^2^ and 520 cell/mm^2^, 2.1-fold increase, respectively) (*p* < 0.001) (Fig. 4d). As internal experimental controls, dishes pre-coated with CO and consecutive slices of the same LN were used to ensure that both experimental groups were seeded at the same number and to minimize regional variations in ECM composition of each lymph node, which in turn can dictate the cell adhesion rates.

To extend these observations to another model, we transfected non-metastatic and poor invasive T47D breast cancer cells with control and specific NDRG4-shRNAs (Supplementary Fig. S4). Polyclonal NDRG4-silenced T47D populations showed increased adhesion to LN sections relative to control cells (294 cells/mm^2^ and 67 cell/mm^2^, 4.4-fold increase, respectively) (*p* = 0.008) (Fig. 4e). Our results show that NDRG4 knockdown in both breast cancer cell lines significantly increases LN tumor cell adhesion.

Next, to assess the mechanism by which NDRG4 silencing promotes lymphatic adhesion, we performed in vitro adhesion assays using MCF-7 cells and distinct purified ECM components present in lymph nodes, including VN, FN, and CO.^28,29^ Our results showed a significant change in the adhesive properties (‘adhesive switch’) of MCF-7 cells after NDRG4 silencing, with a 31% increase in cell adhesion to VN (*p* = 0.02) and a concomitant 39% decrease in cell adhesion to FN (*p* = 0.002). The adhesive switch from FN adhesion to VN adhesion is specific since similar results were not observed for CO adhesion (Fig. 4f).

Given that cell adhesion is the basis for cell mobility and spreading, we next investigated whether NDRG4 silencing impacted in vitro tumor cell haptotactic migration towards ECM ligands. We noticed that NDRG4 silencing enhanced MCF-7 cell migration towards VN (2.2-fold increase, *p* = 0.0006), with no significant impairment on migration towards FN (0.75-fold decrease, *p* < 0.05) (Fig. 4g). In agreement, following NDRG4 knockdown T47D cells also showed an increased migration towards VN (3.7-fold increase, *p* = 0.02) (Fig. 4h). These results indicate that NDRG4 gene silencing enhances migration towards VN in both non-metastatic breast tumor cell lines.

### NDRG4 controls β1 integrin clustering at cell membrane

Among the cell receptors associated with the tripeptide Arg-Gly-Asp (RGD) ligands, αv-integrin subunits are well known FN and VN receptors that form complexes with five different integrin β subunits (β1, β3, β5, β6, and β8).^30^ Considering that MCF-7 cells constitutively express αvβ1 and αvβ5 integrins, but do not express αvβ3, αvβ6 or αvβ8 and also that αv co-immunoprecipitates with β1 (confirming that αvβ1 heterodimers are alternative VN receptors in MCF-7 cells)^31–33^ and Supplementary Fig. S5, we focused our subsequent analysis on the putative role of β1 and β5 integrins in NDRG4-mediated migration.

**Fig. 5:**
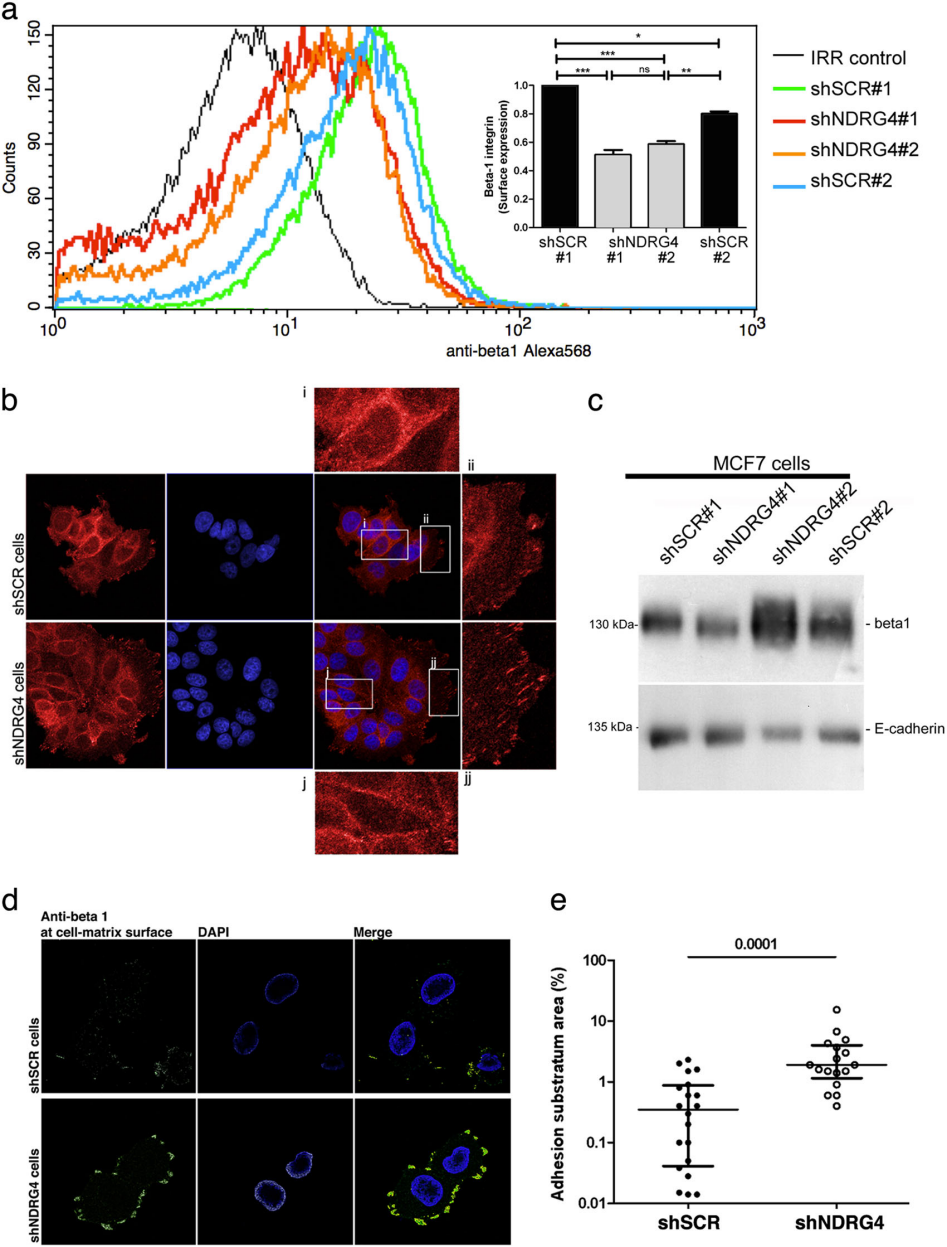
NDRG4 knockdown promotes clustering of β1-integrin at cell surface of MCF-7 adherent cells. **a** Representative of flow citometry histograms using anti-β1 integrin monoclonal antibody (MAB1965) and irrelevant IgG as negative control (IRR). Quantification of the flow cytometry analysis of β1 integrin expression levels at the cell surface of nonadherent MCF-7 shNDRG4 or shSCR cells. Error bars represent SEM of biological replicates (*n* = 3). *p < 0.01, **p < 0.01, ***p < 0.001, ns = not significant by one-way ANOVA (insert). **b** Confocal microscopy images of β1 integrin subunit (MAB1965, red) at the surface of VN-adherent MCF-7 shNDRG4 or shSCR cells. **c** Western blot analysis of β1 integrin subunits and E-cadherin (as loading control) from MCF-7 control (shSCR) or NDRG4-depleted cells (shNDRG4). **d** Confocal images at the level of the ventral cell surface of individual MCF-7 shNDRG4 (*n* = 17) or shSCR (*n* = 20) cells showing different levels of β1 integrin clustering at cell-matrix interface. **e** Quantification of images in (**d**) showing the percentage of ventral cell surface covered by clusters of b1 integrin. Each dot represents an individual cell. ***p < 0.001 by two-tailed *t* tests

**Fig. 6:**
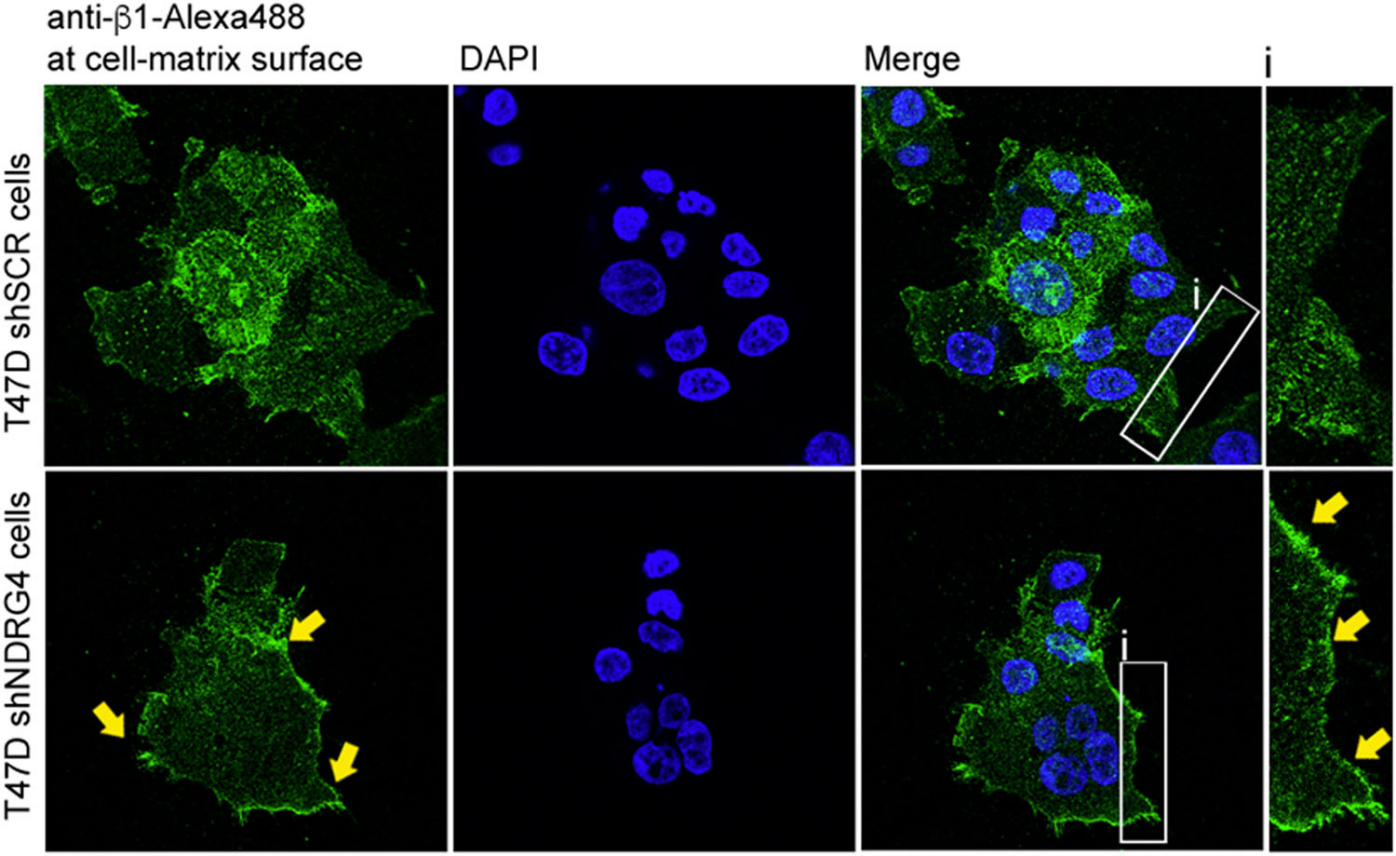
NDRG4 knockdown promotes clustering of β1-integrin at the leading edge of T47D cells. Representative confocal images of β1 integrin subunit (MAB1965, green) at the ventral cell surface of VN-adherent T47D shNDRG4 or shSCR cells

Flow cytometry analysis showed a specific 50% reduction in β1 integrin present on the cell surface of nonadherent shNDRG4 cells in comparison to control cells (Fig. 5a, *p* < 0.01). Given the critical importance of the cell attachment for integrin expression, clustering and biological function,^34^ we next analyzed the αv and β1 distribution at the cell surface of VN-adherent cells using vertical confocal slices. In general, we found that β1-integrin staining was concentrated in cell–cell contacts (Fig. 5b, i, j) and at the leading edge of the cell in MCF-7 cells (Fig. 5b, ii–jj). Surprisingly, z-stacked images taken specifically at the level of the ventral cell surface showed a 5.4-fold increase in the size of β1-integrin clusters in shNDRG4 cells in comparison to control cells (Fig. 5d, e, *p* < 0.001), without altered their total expression levels (Fig. 5c). No differences were observed for αv staining (data not shown). Interestingly, following NDRG4-knockdown, at ventral surfaces of T47D VN-adherent cells β1-integrin clusters did not increased in size, but become more intense in membrane ruffles at the cellular leading edge (lamellipodia protrusions, arrows in Fig. 6).

**Fig. 7:**
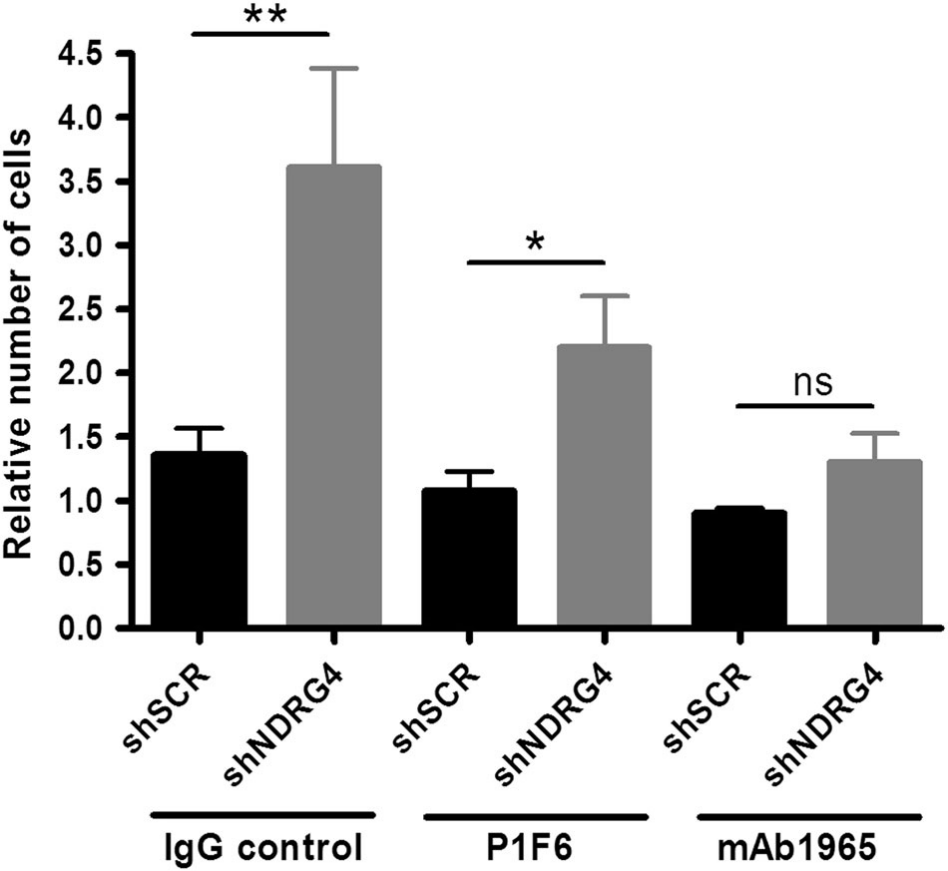
NDRG4 knockdown enhances β1 integrin-mediated cell migration towards vitronectin (VN). Migration to VN (10 μg/ml) is specifically inhibited by the anti-β1 integrin monoclonal antibody (MAB1965) with function-blocking activity. Cells incubated with the anti-αvβ5 integrin monoclonal antibody (clone P1F6) with function-blocking activity and cells incubated with irrelevant IgG were used as controls Error bars represent SEM of biological replicates (*n* = 3). *p < 0.05, **p < 0.01, ns = not significant by two-tailed *t* tests

Finally, inhibition studies using blocking antibodies against αvβ5-integrin (clone P1F6) and β1-integrins (MAB1965) showed that β1-specific inhibition reduced cell motility towards VN by 15% in MCF-7 control cells and by 49% in shNDRG4 cells (Fig. 7, *p* < 0.05). Of note, no significant differences in cell motility inhibition towards VN were observed when shNDRG4 and control cells were incubated with an anti-αvβ5-integrin blocking antibody. Collectively, these results indicate that NDRG4 controls β1-integrin clustering at the cell-matrix interface and determines the migratory behavior of breast cancer cells towards VN.

## Discussion

NDRG4 is a highly expressed gene in the brain (NDRG4-B and B^var^) and heart (NDRG4-H) and a potential tumor suppressor in different types of tumors.^35^ Recent reports revealed that epigenetic mechanisms are especially important in controlling NDRG4 expression, as treatments with DNA methyltransferase and/or histone deacetylase inhibitors have been shown to induce the reactivation of NDRG4 in colorectal,^16,36,37^ gastric^38^ and pancreatic cancers.^39^ Clinically, the detection of NDRG4 methylation in fecal DNA is part of a multitarget FDA-approved test for the non-invasive detection of colorectal cancer (“Cologuard”).^22,39–41^ NDRG4 hypermethylation has also been detected in pancreatic secretions from patients with pancreatic cancer.^42^ Furthermore, multivariate analysis showed that NDRG4 expression was an independent prognostic factor in glioblastoma (GBM) patients and that reduced NDRG4 expression increased the likelihood of dying from GBM by three-fold. However, NDRG4 silencing in GBM does not appear to be related to DNA promoter hypermethylation.^43^

In this study, we provided evidence that NDRG4 is a mechanistic biomarker in breast cancer patients that actively confers distinct metastatic advantages to tumor cells.

While NDRG4 downregulation is functionally associated with enhanced lymph node affinity and cell mobility in two non-metastatic and poor invasive breast cancer cell lines, NDRG4 DNA methylation is predictive for the development of metastatic disease. Mechanistic biomarkers for cancer are potentially more valuable than those that are byproducts of tumor progression (descriptive biomarkers) in differential diagnosis, tumor stratification, and target therapy.^44^

As a biomarker, we demonstrated that NDRG4 DNA hypermethylation is associated with negative prognostic factors, such as tumor size, p53 overexpression and the presence of lymph node metastasis, in breast cancer patients. Most importantly, we demonstrated that NDRG4 hypermethylation is associated with a lower 5-year distant metastases-free survival rate. Evidence for functional association between lymph node metastasis and NDRG4 depletion was also observed in vitro, when NDRG4 knockdown contributed significantly to tumor cell adhesion to frozen lymph node sections—a correlate with lymphatic metastasis (Fig. 4d, e). This is interesting in light of observations that adhesion to frozen lymph node sections is enhanced in metastatic breast cancer cells lines in comparison to non-metastatic cells and normal breast epithelial cells.^45^ Changes in lymph node adhesiveness are mediated primarily by specific changes in integrin expression at the cell surface. Integrins are cell surface receptors that determine tumor cell mobility and survival in secondary organs.^46^ For instance, α3β1-integrin expression mediates breast carcinoma cell adhesion to FN on lymph nodes,^47^ and αvβ3-integrin mediates melanoma cell adhesion to VN in the lymph node.^48^ Together, these studies indicate that the gain-of-function expression of different integrins may direct metastasis to the same secondary organ.

In this context, we also observed that NDRG4 knockdown promotes an “adhesive switch” by decreasing cell adhesion to FN and increasing cell adhesion and migration towards VN, an important component of human lymph nodes. VN is a glycoprotein that is synthesized primarily by liver cells and is a known adhesive substrate for cells expressing at least one of four known receptors: αvβ1, αvβ3, αvβ5 or αIIbβ3 integrins. Integrin-VN binding exerts a determinant function on tumor growth and metastasis in a variety of human tumors.^49,50^ VN occurs in the blood plasma at relatively higher concentrations and is deposited extravascularly in many tissues, particularly in reactive stroma and in areas of fibrosis, as observed in lymph nodes infiltrated by metastatic carcinoma cells.^29,51^

Of note, NDRG4 expression emerged in this study within a group of epigenetically silenced genes in breast cancer cells that were significantly associated with integrin pathways (e.g., ITGA5 and COL9A3). In this context, we also demonstrated that, exclusively in adherent conditions, NDRG4 silencing modulates integrin signaling by assembling β1-integrins into large punctate clusters at the cell-matrix interface and at the lamellipodia protrusions (Figs. 5 and 6), which were barely observed in NDRG4 control cells. Previous studies have shown that integrin heterodimers assemble laterally at the cell surface and form large adhesive plaques that regulate signal transduction and increase metastatic potential.^52^ In suspension, depletion of NDRG4 seems to accelerate integrin internalization compared to control cells (Fig. 5a). Although too speculative at this stage, a possible explanation is that NDRG4 controls β1 integrin recycling in breast cancer cells. In agreement with this assumption, Benesh and colleagues^13^ demonstrated that NDRG4 is required for recycling endosomes at the basal surface of migrating epicardial cells, thus linking important pathways that regulate recycling of integrins (endocytic routes) with the proposed function of NDRG4 in breast cancer. Interestingly, the mechanistic role of NDRG4 in the clustering of β1-integrins in breast tumor cells resembles its role in Na_v_ channel clustering in the Peripheral nervous system (PNS) and CNS.^11^

Integrin β1 is the most promiscuous integrin β-subunit. Integrin β1 is found in 12 different integrin heterodimers and, in normal mammary ducts, the protein is found in heterodimers with α1, α2, α3, α5 and α6 and mediates adhesion to collagens (e.g., α1β1, α2β1), laminin (e.g., α6β1) and RGD-containing ligands (e.g., α3β1, α5β1).^53^ Several studies have provided insight into the critical role of β1 integrin during normal mammary gland development and in the malignant transformation of breast tumor cells.^27,54^ Consistent with our data, β1 integrin is not required for tumor initiation but is critical during malignant progression and metastatic spread in the MMTV-Neu-NDL2–4 mouse model.^55^

Taken together, our functional and clinical observations imply a causal relationship between NDRG4 status and the aggressive biological behavior of ductal invasive breast cancers, suggesting that NDRG4 is a potential mechanistic biomarker that is functionally associated with metastatic disease.

## Methods

### Patient samples

Invasive breast tumors were obtained from 61 consecutively treated patients at the A.C. Camargo Cancer Center (ACC) from 1998 to 2001. Normal breast samples were obtained from women undergoing reduction mammoplasty. All participants signed written informed consent. Patients younger than 35 years, with multiple cancer diagnoses or with hereditary breast cancer, or if they received neoadjuvant radio/chemotherapy were excluded from this study. This study was approved by the Brazilian National and by ACC Ethics Committees (728/2000, 1357/10).

### Cell lines and 5Aza-dC treatment

Tumor cell lines (MCF-7, MDA-MB231, and MDA-MB435) with different tumorigenic and metastatic characteristics were commercially available from ATCC. T47D cells were a gift from F. Pittella (University of Brasilia). The cell lines were cultured in RPMI 1640 medium (Sigma-Aldrich, Darmstadt, Germany) supplemented with 10% fetal calf serum (FCS), 1% L-glutamine, and 1% penicillin/streptomycin, at 37 °C and 5% CO_2_. The cells tested negative for mycoplasma using PCR-based detection and were authenticated using short tandem repeat (STR) profiling. The cells were treated for 6 days with freshly prepared 1 μM 5Aza-dC (Sigma-Aldrich).

### RNA and DNA extraction

Total RNA was isolated using TRIzol (Invitrogen) according to the manufacturer’s instructions. RNA integrity was evaluated using the Agilent 2100-Bioanalyzer. For real-time PCR analysis, 5 μg of total RNA were treated with RQ1 RNase-free DNase (Promega) according to the manufacturer’s instructions. Genomic DNA from tumor cell lines and primary tumors was extracted via standard phenol/chloroform extraction. DNA quality was verified via electrophoresis on an agarose gel stained with ethidium bromide.

### Microarray analysis

Total RNA was purified using CsCl gradient and a two-round RNA amplification procedure was performed as described previously.^56^ The amplified RNA was used in a reverse transcription reaction in the presence of random hexamer primers (Invitrogen), Cy3- or Cy5-labeled dCTP (Amersham Biosciences), and SuperScript II (Invitrogen). A customized cDNA platform generated by the Human Cancer Genome Project consisting of 4608 open reading frame expressed sequence tags (ORESTES) that represented different human transcripts was used for gene expression analysis (4.8 K platform).^57^ Equal amounts of Cy-labeled cDNA derived from tumor cell lines (untreated or treated with 5-Aza-2′-deoxycytidine, 5Aza-dC) were mixed with Cy-labeled cDNA obtained from human non-tumor mammalian epithelial cell line HB4a used as reference for all co-hybridizations. Dye swapping was performed, and hybridizations were performed in duplicate, resulting in four independent hybridizations for each cell line. The arrays were scanned and extracted as described previously.^56^ In cDNA microarray data, the differentially expressed genes were determined by comparison of genes that were reactivated by 5Aza-dC treatment in MDA-MB231 cell line (fold > 3).

### cDNA synthesis and real-time PCR

cDNA synthesis was performed with 2 μg of DNA-free RNA using Superscript II Reverse Transcriptase (Invitrogen) and random hexamers. Real-time PCR was performed with Power SYBR Green using an ABI 7500 System (Applied Biosystems). Primers specific for NDRG4 mRNA amplification (Forward 5′ CCT TCC TGG CAG ACT TGA AGA CT 3′ and Reverse 5′ CAG CTT CCC AGT CTG TGT TGG 3′) were designed in different exons using Primer Express 2.0 (Applied Biosystems). The relative quantification of gene expression was performed using the mathematical model developed by Pfaffl.^58^ Untreated cell lines were used as reference samples, and Hydroxymethylbilane synthase (HMBS, Fw 5′ GGC AAT GCG GCT GCA A 3′ and Rev 5′ GGG TAC CCA CGC GAA TCA C 3′) was selected as endogenous control gene.

### CpG island identification and bisulfite sequencing

Genomic DNA was subjected to sodium bisulfite treatment to modify unmethylated cytosine to uracil, as described previously.^59^ The genomic sequence upstream of the NDRG4 gene was analyzed for the presence of a CpG island using the UCSC Genome Browser (http://genome.ucsc.edu/). Bisulfite-treated DNA was amplified via a nested-PCR protocol, using primers (Forward 5′ GGTTTTTTTTGGGAGTTTAAAT 3′ and Reverse 5′ AAACTAACC CTAAACTCAAAAA 3′; Forward2 5′ TTTTGGG AGTTTAAATAAAGATTA 3′ and Reverse2 5′AAAAAAACTAA CCCTAAAATAA 3′) designed to amplify a CpG-rich region located upstream of the NDRG4 gene (−387 to +103 relative to the transcription start site (TSS), encompassing 82 CpG dinucleotides). Hypermethylation in breast tumor cell lines was determined via DNA sequencing after bisulfite modification. The amplified products were cloned using the pGEM-T system (Promega). Five positive clones were sequenced for each cell line using the Big Dye Terminator Cycle Sequencing kit and an ABI3130 sequencer (Applied Biosystems). The methylation percentage for each sample was calculated as the proportion of unconverted CpG dinucleotides among all the CpGs analyzed in all five positive clones.

### Methylation analysis

Hypermethylation in primary breast tumors was determined via nested methylation-specific PCR, and the amplified fragments were analyzed on silver-stained 8% polyacrylamide gels. Briefly, bisulfite-converted DNA was PCR-amplified with the bisulfite sequencing external primers, as described above, and 1 µL of the first reaction was used in the nested reaction with primers specific for methylated (Forward 5′GCGCGGTCGTCGTTTTTC3′ and Reverse 5′AAAACTAACCGAGATGCCACG3′) and unmethylated DNA (Forward 5′AGGTTTTGTGT TGTGGTTTTTGTTT3′ and Reverse 5′AACCAAACTAA AAACAATCCAACA3′). The Chi-square test or Fisher’s exact test was used to examine the association between NDRG4 hypermethylation and clinical-pathological parameters. Disease-specific survival and distant metastases-free survival curves were calculated with the Kaplan–Meier method. The log-rank test was used to assess statistical differences between groups. Multivariate analysis was performed using a Cox proportional hazard model (stepwise forward selection). All variables presenting a *p*-value < 0.20 in the univariate analysis were selected to build a multiple model. For all tests, the type 1 error (α) was established as 0.05, and the results were considered to be statistically significant when *p* < 0.05. Alternatively, DNA methylation and gene expression data from TCGA samples were obtained using the MethHC (http://methhc.mbc.nctu.edu.tw) and MethSurv (https://biit.cs.ut.ee/methsurv/)^24^ online resources. Essentially, survival analysis for the CpG probe cg01466678-NDRG4 (promoter region, chr16:58497395) was conducted in 782 breast cancer patients, dichotomized in higher and lower NDRG4-methylation patient groups.

### NDRG4 knockdown

For stable transfection of NDRG4 shRNA into MCF-7 and T47D breast tumor cells, the pGFP-V-RS vector containing shRNA inserts which targeted the 3′-unttranslated region of NDRG4 (TRCN0000134583 and TRCN0000137216) was purchased (Origene). Tumor cells were transfected using Fugene, according to the manufacturer’s recommendations (Roche). After transfection, the cells were selected with 1 μg/mL of puromycin for 10 days. Whereas, NDRG4-silenced clones were obtained by limiting dilution in MCF-7 cells, we worked with NDRG4-knockdown polyclonal populations in T47D cells. NDRG4 expression was analyzed via real-time PCR and western blot.

### Western blot

Nuclear and cytoplasmic fractions of MCF-7 cells and knockdown clones were extracted with the Protein Extraction NucBuster^TM^ kit (Novagen) according to the manufacturer’s recommendations. Approximately 30 μg of cytoplasmic protein fractions were separated via 12% SDS-PAGE, transferred to a nitrocellulose membrane (Amersham) and immunoblotted with a Prestige® anti-NDRG4 antibody (HPA0153013, Sigma) at 1:400 dilution using Western Blot Detection Kit ECL^TM^ Reagents (GE Healthcare). This polyclonal antibody has been validated for western blotting and specifically recognizes all three isoforms of NDRG4 (H, B, and B^var^).^14^ Actin was used as loading control (anti-actin SIGMA; 1:600 dilution). In each blot, all the samples were derived from the same experiment and gels/blots were processed in parallel.

### Proliferation assay

MCF-7 cells and NDRG4-silenced cells (2 × 10^4^ cells/well) were plated in six-well plates (Costar) and cultured for 8 days. The cells were then trypsinized, stained with 0.4% trypan blue (Invitrogen), and counted daily using a hemocytometer. Three independent assays were performed in duplicate. Statistical analysis was performed by one-way ANOVA followed by Dunn’s multiple comparison test (GraphPad Prism® version 4.03).

### Lymph node adhesion assay

Tumor cell adhesion to fresh lymph nodes was performed as described previously.^60^ Briefly, mesenteric lymph nodes from healthy Wistar rats were extracted and immediately embedded and frozen in OCT compound. Four 5 µm cryostat sections from each lymph node were mounted on cover-slips, gently washed twice with Phosphate-buffered saline (PBS), rehydrated (PBS, 15 min) and blocked (2.5% BSA, 20 min) at 37 °C. Red fluorescent tumor cells were obtained by labeling 10^6^ shNDRG4 and shCtrl breast tumor cells with 2 µM 1,1′-Dioctadecyl-3,3,3′,3′-Tetramethylindo dicarbocyanine Perchlorate (DiIC_18_) cell tracker dye, according to the manufacturer’s instructions (V-22885, Molecular Probes). The labeled tumor cells (10^5^) were plated on lymph node sections and incubated for 90 min at 37 °C. Nonadherent cells were washed, and the remaining cells were fixed with 4% Paraformaldehyde (PFA) for 15 min. The quantification of cell density (cells/mm^2^) was achieved by using a ×10 objective and counting the number of red fluorescent tumor cells per lymph node area in 20 different optical fields. Dishes pre-coated with collagen type-1 (CO) were used as a control to ensure that both experimental groups were seeded at the same number. All images were analyzed using ImageJ (NIH, Bethesda, MD).

### Migration assay

Haptotactic migration assays were performed in Transwell (Corning) plates with 8 μm polycarbonate filters coated with 10 μg/mL of VN, FN or CO for 2 h at 37 °C and blocked with 2.5% bovine serum albumin in RPMI 1640. Breast tumor shNDRG4 or control cells were seeded in serum-free RPMI 1640 in duplicate (10^5^ per well) and allowed to migrate for 24 h at 37 °C and 5% CO_2_. The top side of the filters was scraped with cotton swabs, fixed and stained with DAPI (4′,6-diamidino-2-phenylindole). Nuclei were counted at a ×100 magnification in 20 different optical fields. Three or four independent assays were performed in duplicate. Statistical analysis was performed by one-way ANOVA followed by Tukey’s Multiple Comparison Test (GraphPad Prism® versão 4.03).

### Gene expression-based outcome for breast cancer online (GOBO) analysis

Expression data for different breast cancer sub-types (gene set analysis tumors) were obtained using the Lund University online resource at http://co.bmc.lu.se/gobo/gsa.pl.^26^

### Immunohistochemistry

Orthotopic xenografted tumors were harvested, fixed in buffered formalin and embedded in paraffin. Sections were heated at 60 °C for 20 min and deparaffinized in xylene, followed by a graded series of alcohol (100–75%), and rehydrated in water. The slides were placed in 3% hydrogen peroxide three times for 5 min each to quench endogenous peroxidase and then blocked for avidin/biotin (Biotin Blocking System; DAKO) and protein (Protein Block Serum-Free; DAKO) prior to incubation for 24 h with the primary anti-NDRG4 antibody (Sigma, HPA015313) diluted 1:500. The slides were washed in PBS, incubated with biotinylated goat anti-mouse IgG for 20 min and then incubated with a streptavidin-biotin peroxidase LSAB kit (Dako). The immunostaining was performed by incubating slides in a diaminobenzidine (Dako) solution containing 1 µL of chromogen per 50 µL of buffer substrate for 5 min. After chromogen development, the slides were washed, dehydrated with alcohol and xylene, and mounted with coverslips using a permanent mounting medium.

### Immunofluorescence and image processing

Monolayers of MCF-7 or T47D cells grown to 50–60% confluence on 8-well slides (Labtek, Campbell) were fixed in 4% formaldehyde for 10 min, washed in PBS and blocked with 0.1% Triton-X, 2% BSA, and 1% FBS for 15 min at 4 °C. The cells were incubated with an anti-β1 monoclonal antibody (MAB1965, Millipore) for 1 h at room temperature. Detection was performed with Alexa Fluor-labeled secondary antibodies (Molecular Probes), and z-stack tridimensional (3D) images were collected on an inverted confocal microscope (Leica TCS-SP8, Germany) at the National Institute of Pharmacology and Molecular Biology of the Federal University of São Paulo (Infar/Unifesp). Image processing and the quantification of β1-integrin cluster sizes on the ventral surface of MCF-7 cells were performed using ImageJ (NIH, Bethesda, MD) and normalized against the total surface area of the same cell, as described for focal adhesion quantification by Horzum et al.^61^

### Reporting Summary

Further information on experimental design is available in the Nature Research Reporting Summary linked to this article.

## Supplementary information

Supplementary Figures

Supplementary Data 1

Supplementary Data 2

Supplementary Data 3

Supplementary Data 4

Reporting Summary

## Data Availability

The data generated and analyzed during this study are described in the following data record: 10.6084/m9.figshare.c.4364174. The accession number for the raw and processed data from the cDNA microarray experiments reported in this paper is NCBI GEO: GSE127885 http://identifiers.org/geo:GSE127885. Methylation, expression level and clinical data files are available in the figshare repository: 10.6084/m9.figshare.7553657. Data files that support other findings, figures and tables are available on request as described at 10.6084/m9.figshare.7578404. Uncropped blots are also available as part of supplementary information (Supplementary Figs. 5–6).

## Supplementary information

**Supplementary information** accompanies the paper on the *npj Breast Cancer* website (10.1038/s41523-019-0106-x).

## References

[1] Steeg PS (2016). Targeting metastasis. Nat. Rev. Cancer.

[2] Tevaarwerk AJ (2013). Survival in patients with metastatic recurrent breast cancer after adjuvant chemotherapy: little evidence of improvement over the past 30 years. Cancer.

[3] Paget S (1889). The distribution of secondary growths in cancer of the breast. Lancet.

[4] LaDuca H (2014). Utilization of multigene panels in hereditary cancer predisposition testing: analysis of more than 2,000 patients. Genet Med..

[5] Couch FJ (2017). Associations between cancer predisposition testing panel genes and breast cancer. JAMA Oncol..

[6] Patel, S. A. & Vanharanta, S. Epigenetic determinants of metastasis. *Mol. Oncol.* 8. 10.1016/j.molonc.2016.09.008 (2016). 10.1016/j.molonc.2016.09.008 PMC5423227 27756687

[7] Fang F (2011). Breast cancer methylomes establish an epigenomic foundation for metastasis. Sci. Transl. Med.

[8] Reyngold M (2014). Remodeling of the methylation landscape in breast cancer metastasis. PLoS ONE.

[9] Zhou RH (2001). Characterization of the Human NDRG Gene Family: a newly identified member, NDRG4, is specifically expressed in brain and heart. Genomics.

[10] Yamamoto H (2011). NDRG4 protein-deficient mice exhibit spatial learning deficits and vulnerabilities to cerebral ischemia. J. Biol. Chem..

[11] Fontenas L (2016). Neuronal Ndrg4 is essential for nodes of Ranvier Organization in Zebrafish. PLoS Genet..

[12] Qu X (2008). Ndrg4 is required for normal myocyte proliferation during early cardiac development in zebrafish. Dev. Biol..

[13] Benesh EC (2013). Bves and NDRG4 regulate directional epicardial cell migration through autocrine extracellular matrix deposition. Mol. Biol. Cell.

[14] Schilling SH (2009). NDRG4 is required for cell cycle progression and survival in glioblastoma cells. J. Biol. Chem..

[15] Ding W (2012). NDRG4 is down-regulated in glioblastoma and inhibits cell proliferation. OMICS.

[16] Melotte V (2009). N-Myc downstream-regulated gene 4 (NDRG4): A candidate tumor suppressor gene and potential biomarker for colorectal cancer.. J. Natl. Cancer Inst..

[17] Jandrey, E. et al. (2019): Collection holding methylation, expression level and clinical data related to NDRG4 promoter hypermethylation: a mechanistic biomarker associated with metastatic progression in breast cancer patients. figshare. *Fileset*. 10.6084/m9.figshare.c.4364174 (2019). 10.1038/s41523-019-0106-x PMC6450950 30963110

[18] Subramanian A (2005). Gene set enrichment analysis: A knowledge-based approach for interpreting genome-wide expression profiles. Proc. Natl Acad. Sci. USA.

[19] Liberzon A (2011). Molecular signatures database (MSigDB) 3.0. Bioinformatics.

[20] Felding-Habermann B (2003). Integrin adhesion receptors in tumor metastasis. Clin. Exp. Metastas..

[21] Chen PS (2007). CTGF enhances the motility of breast cancer cells via an integrin alphav beta3 ERK1/2-dependent S100A4-upregulated pathway. J. Cell Sci..

[22] Imperiale TF (2014). Multitarget stool DNA testing for colorectal-cancer screening. N. Engl. J. Med..

[23] Huang WY (2015). MethHC: a database of DNA methylation and gene expression in human cancer. Nucleic Acids Res..

[24] Modhukur M (2018). MethSurv: a web tool to perform multivariable survival analysis using DNA methylation data. Epigenomics.

[25] Neve RM (2006). A collection of breast cancer cell lines for the study of functionally distinct cancer subtypes. Cancer Cell.

[26] Ringner M, Fredlund E, Hakkinen J, Borg A, Staaf J (2011). GOBO: Gene Expression-Based Outcome for Breast Cancer Online. PLoS ONE.

[27] Rizwan, A., Cheng, M., Bhujwalla, Z. M., Krishnamachary, B. & Jiang, L. Breast cancer cell adhesome and degradome interact to drive metastasis. *NPJ Breast Cancer* **28**. 10.1038/npjbcancer.2015.17 (2015). 10.1038/npjbcancer.2015.17 PMC5515192 28721370

[28] Kramer RH, Rose SD, McDonald KA (1988). Basement membrane components associated with the extracellular matrix of the lymphnodes. Cell Tissue Res..

[29] Reily JT, Nash JRG (1988). Vitronectin (serum spreading factor): its localizations in normal and fibrotic tissue. J. Clin. Pathol..

[30] Plow EF, Haas TA, Zhang L, Loftus J, Smith JW (2000). Ligand binding to integrins. J. Biol. Chem..

[31] Taherian A, Li X, Liu Y, Haas TA (2011). Differences in integrin expression and signaling within human breast cancer cells. BMC Cancer.

[32] Brooks PC (1997). Insulin-like growth factor receptor cooperates with integrin αvβ5 to promote tumor cell dissemination in vivo. J. Clin. Invest..

[33] Meyer T, Marshall JF, Hart IR (1998). Expression of αv integrins and vitronectin receptor identity in breast cancer cells. Br. J. Cancer.

[34] Toscani AM (2017). Distinct ErbB2 receptor populations differentially interact with beta1 integrin in breast cancer cell models. PLoS ONE.

[35] Geleta B, Makonnen E, Abay SM (2016). N-myc downstream regulated gene (NDRG): role in cancer metastasis suppression and as drug target in cancer therapeutics. J. Cancer Sci. Ther..

[36] Xiao W (2015). Quantitative detection of methylated NDRG4 gene as a candidate biomarker for diagnosis of colorectal cancer. Oncol. Lett..

[37] Kadiyska T, Nossikoff A (2015). Stool DNA methylation assays in colorectal cancer screening. World J. Gastroenterol..

[38] Chen X (2017). NDRG4 hypermethylation is a potential biomarker for diagnosis and prognosis of gastric cancer in Chinese population. Oncotarget.

[39] Kisiel JB (2012). Stool DNA testing for the detection of pancreatic cancer: assessment of methylation marker candidates. Cancer.

[40] Lu H, Huang S, Zhang X, Wang D, Zhang X (2014). DNA methylation analysis of SFRP2, GATA4/5, NDRG4 and VIM for the detection of colorectal cancer in fecal DNA. Oncol. Lett..

[41] A Stool DNA Test. (2014). (Cologuard) for Colorectal Cancer Screening. J. Am. Med. Assoc..

[42] Kisiel JB (2015). New DNA methylation markers for pancreatic cancer: discovery, tissue validation, and pilot testing in pancreatic juice. Clin. Cancer Res..

[43] Li S, Yang B, Li G, He S, Li Y (2013). Downregulation of N-Myc downstream-regulated gene 4 influences patient survival in gliomas. Brain Tumor Pathol..

[44] Robinson WH (2013). Mechanistic biomarkers for clinical decision making in rheumatic diseases. Nat. Rev. Rheumatol..

[45] Brodt P (1990). Tumor cell adhesion to frozen lymph node sections - a correlate of lymphatic metastasis in breast carcinoma models of human and rat origin. Breast Cancer Res. Treat..

[46] Desgrosellier JS, Cheresh DA (2010). Integrins in cancer: biological implications and therapeutic opportunities. Nat. Rev. Cancer.

[47] Tawil NJ (1996). Integrin alpha-3/beta-1 can promote adhesion and spreading of metastatic breast carcinoma cells on the lymph node stroma. Int. J. Cancer.

[48] Nip J, Shibata H, Loskutoff DJ, Cheresh D, Brodt P (1992). Human mela- noma cells derived from lymphatic metastases use integrin αvβ3 to adhere to lymph node vitronectin. J. Clin. Invest..

[49] Schneider G (2016). Evidence that vitronectin is a potent migration-enhancing factor for cancer cells chaperoned by fibrinogen: a novel view of the metastasis of cancer cells to low-fibrinogen lymphatics and body cavities. Oncotarget.

[50] Schneider G (2016). Vitronectin in the ascites of human ovarian carcinoma acts as a potent chemoattractant for ovarian carcinoma: Implication for metastasis by cancer stem cells. J. Cancer Stem Cell Res.

[51] Aaboea M, Offersen BV, Christensen A, Andreasen PA (2003). Vitr. Human Breast Carcinomas Bioch Biophys. Acta.

[52] Wennerberg K (2000). The cytoplasmic tyrosines of integrin subunit β1 are involved in focal adhesion kinase activation. Mol. Cell Biol..

[53] Taddei I (2003). Integrins in mammary gland development and differentiation of mammary epithelium. J. Mammary Gland Biol. Neoplasia.

[54] Lahlou H, Muller WJ (2011). β1-integrins signaling and mammary tumor progression in transgenic mouse models: implications for human breast cancer. Breast Cancer Res..

[55] Huck L, Pontier SM, Zuo DM, Muller WJ (2010). Beta1-integrin is dispensable for the induction of ErbB2 mammary tumors but plays a critical role in the metastatic phase of tumor progression. Proc. Natl Acad. Sci. USA.

[56] Maschietto M (2008). Molecular profiling of isolated histological components of wilms tumor implicates a common role for the Wnt signaling pathway in kidney and tumor development.. Oncology.

[57] Brentani RR (2005). Gene expression arrays in cancer research: methods and applications. Crit. Rev. Oncol. Hematol..

[58] Pfaffl MW (2001). A new mathematical model for relative quantification in real-time RT-PCR. Nucleic Acids Res.

[59] Goldenberg D (2004). Intraoperative molecular margin analysis in head and neck cancer.. Arch. Otolaryngol. Head Neck Surg..

[60] Brodt P (1989). Tumor cell adhesion to frozen lymph node sectionsman in vitro correlate of lymphatic metastasis.. Clin. Exp. Metastas..

[61] Horzum U, Ozdil B, Pesen-Okvur D (2014). Step-by-step quantitative analysis of focal adhesions. MethodsX.

