# Supplementary material for: NDRG4 promoter hypermethylation is a mechanistic biomarker associated with metastatic progression in breast cancer patients
Source: NPJ Breast Cancer. 2019 Apr 5;5:11. doi: 10.1038/s41523-019-0106-x (PMC6450950; doi:10.1038/s41523-019-0106-x)
Supplement: Supplementary file 1 — Supplementary Figures [file 41523_2019_106_MOESM1_ESM.pdf]

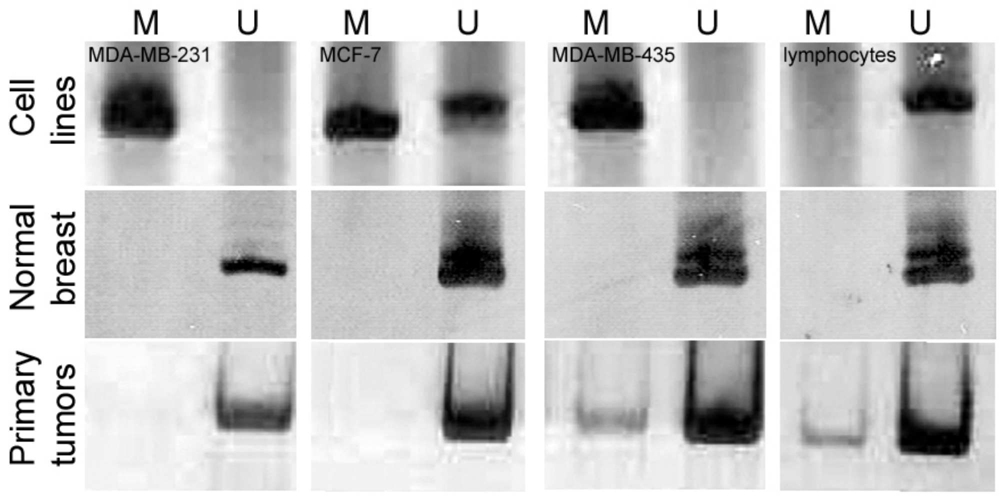

Supplementary Figure S1. Representative results of nested-MSP analysis in breast cancer patients, normal breast and breast cancer cell lines.

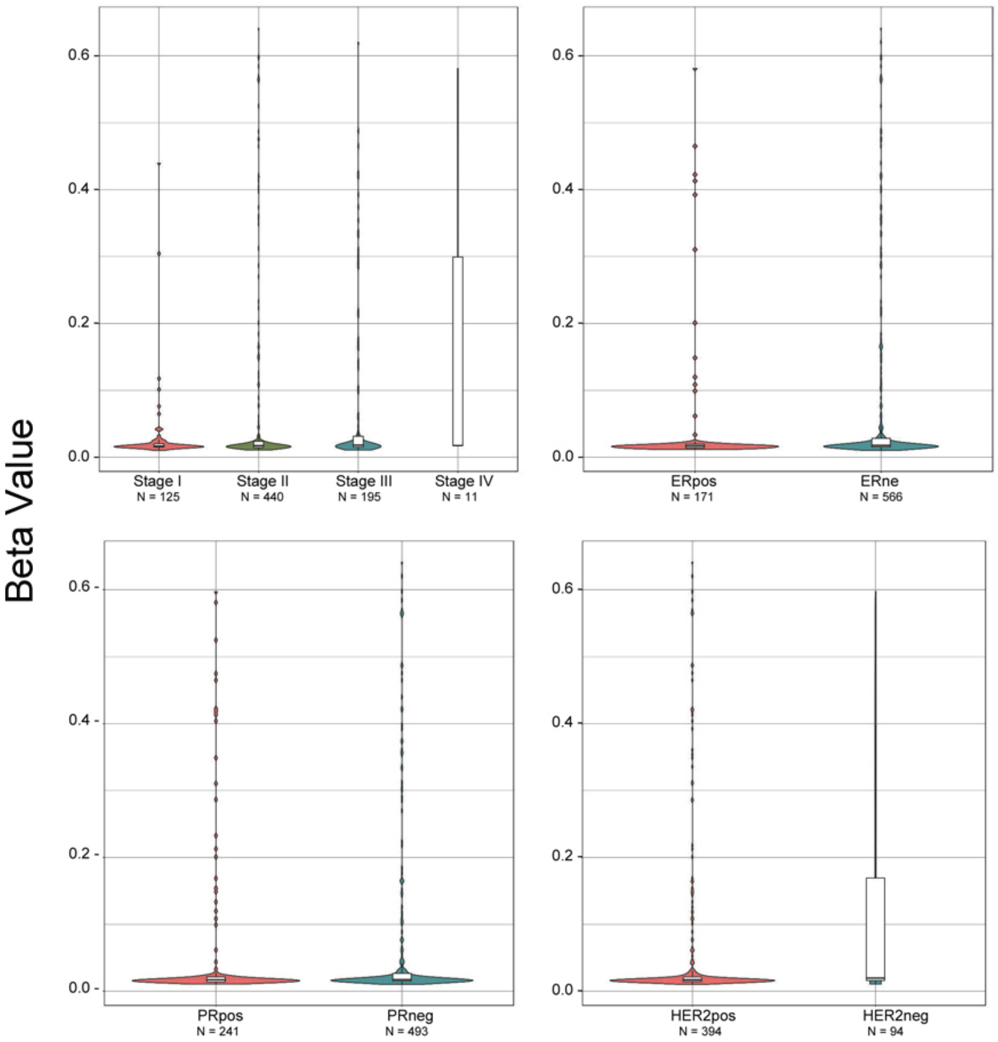

Supplementary Figure S2. Violin plots showing NDRG4 methylation levels for cg01466678 (NDRG4 promoter CpG island) among stage I, II, III and IV

(upper, left) or histological subgroups: ER+ (upper, right), PR+ (lower, left) and HER2+ (lower, right) in breast primary tumors. The X-axis denotes the patient group, while the Y-axis denotes the methylation β-values. Data obtained from MethSurv.

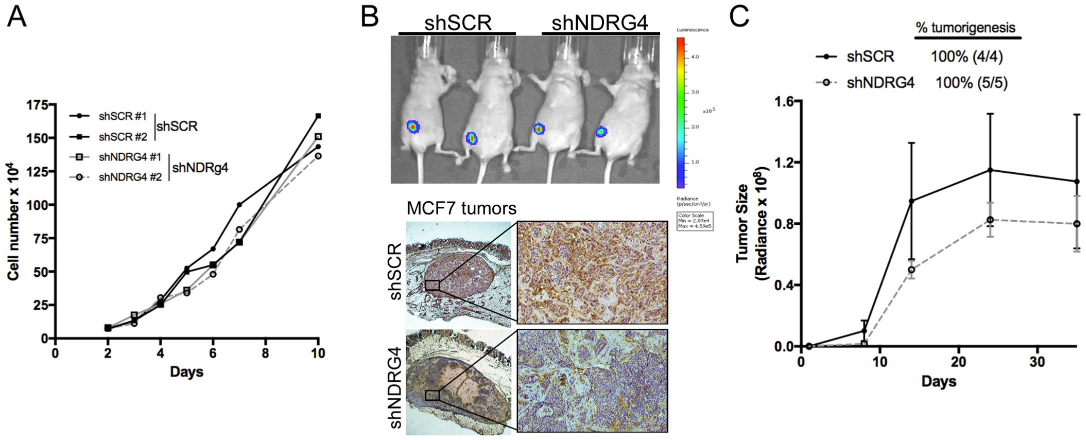

Supplementary Figure S3. (A) Proliferation curves of MCF-7 shNDRG4 and shSCR cells. Proliferation assays were executed by counting the number of viable cells during 10 days after seeding 8,000 cells/well. (B) Representative images of MCF-7 shNDRG4 and shSCR ortothopic tumors in nude mice by bioluminescent intensity with IVIS 200 imaging system (upper) and by immunohistochemistry staining of NDRG4 protein carried out using the polyclonal antibody anti-NDRG4 (HPA0153013) (lower). These cells were engineered with a luciferase gene. (C) Tumor growth kinectics of MCF-7 shNDRG4 and shSCR cells in the m.f.p. by bioluminescent intensity with IVIS 200 imaging system.

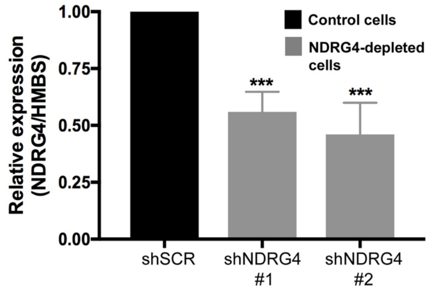

Supplementary Figure S4. RT-qPCR analysis of NDRG4 mRNA expression levels in polyclonal T47D populations transfected with two NDRG4 shRNAs (shNDRG4#1 and shNDRG4#2) or shRNA scramble control (shSCR). Expression levels relative to respective wild type cells and normalized to hydroxymethylbilane synthase (HMBS) endogenous control gene are shown. Error bars represent SEM of

biological replicates (n = 3). ***P < 0.001, ns = not significant, by one-way ANOVA.

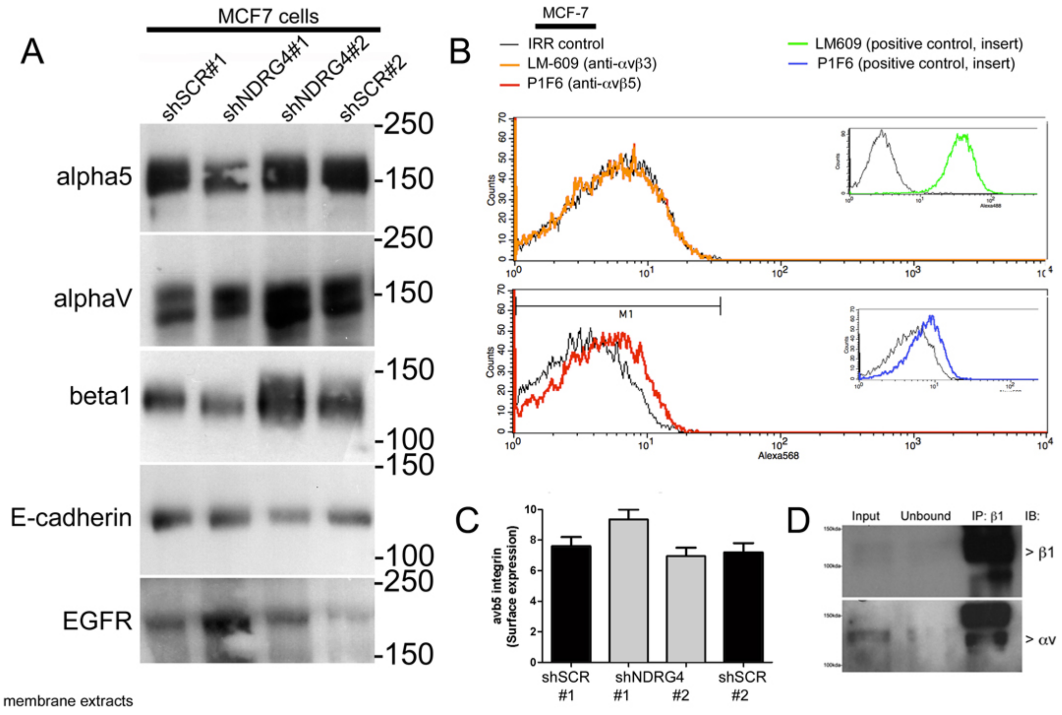

Supplementary Figure S5. (A) Western blot analysis of α5, αv and β1 integrin subunits, e-cadherin and EGFR expression levels in membrane extracts from MCF-7 control (shSCR) or NDRG4-depleted cells (shNDRG4). (B) Flow citometry analysis of αvβ3 and αvβ5 integrins expression at cell surface of MCF-7 wild-type cells. (C) quantification of the flow cytometry analysis of αvβ5 integrin expression levels at cell surface of MCF-7 shSCR or shNDRG4 cells. (D) Immunoprecipitation of β1 integrin subunit (MAB1965) followed by immunoblot analyses of αv integrin subunit (AB1930) from MCF-7 wild-type extracts.

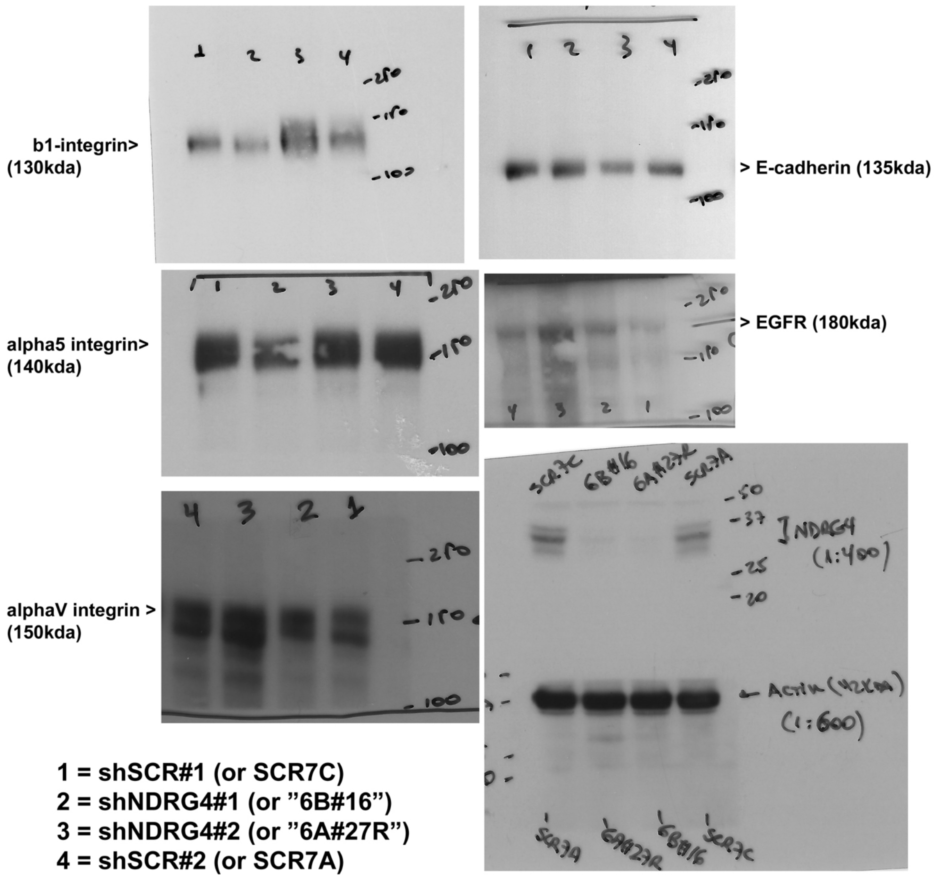

Supplementary Figure S6. Full blot images for Supplementary Figure S5A and Figure 4.

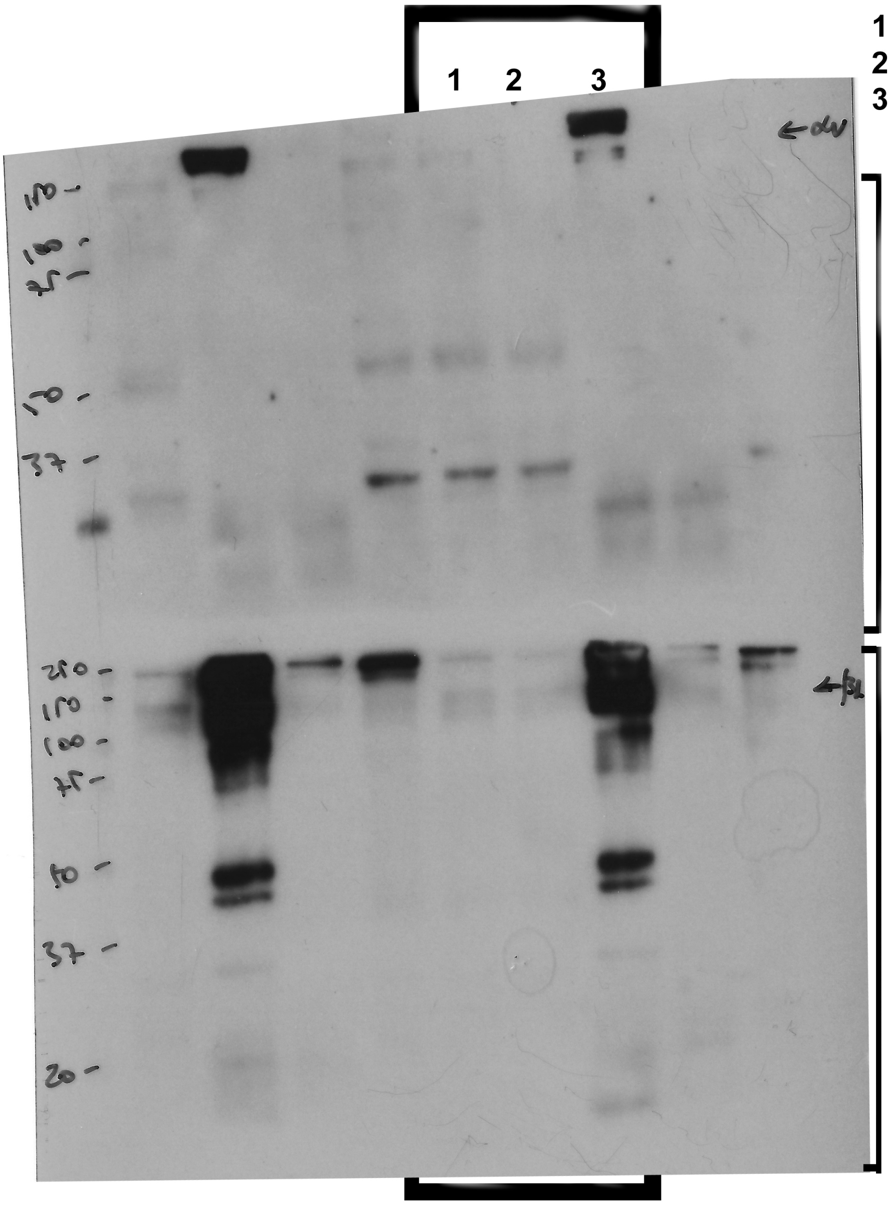

Supplementary Figure S7. Full blot images for Supplementary Figure S5D.
